# Sex Differences in Behavioral Symptoms and the Levels of Circulating GFAP, Tau, and NfL in Patients With Traumatic Brain Injury

**DOI:** 10.3389/fphar.2021.746491

**Published:** 2021-11-26

**Authors:** Dilorom Sass, Vivian A. Guedes, Ethan G. Smith, Rany Vorn, Christina Devoto, Katie A. Edwards, Sara Mithani, James Hentig, Chen Lai, Chelsea Wagner, Kerri Dunbar, David R. Hyde, Leorey Saligan, Michael J. Roy, Jessica Gill

**Affiliations:** ^1^ National Institutes of Nursing Research, NIH, Bethesda, MD, United States; ^2^ Henry M. Jackson Foundation, Bethesda, MD, United States; ^3^ Department of Biological Sciences, Notre Dame, IN, United States; ^4^ Center for Stem Cells and Regenerative Medicine, Galvin Life Sciences, University of Notre Dame, Notre Dame, IN, United States; ^5^ Center for Neuroscience and Regenerative Medicine, Rockville, MD, United States; ^6^ Department of Medicine, Uniformed Services University of the Health Sciences, Bethesda, MD, United States

**Keywords:** biomarkers, traumatic brain injury, sex, behavioral symptoms, gender

## Abstract

Traumatic brain injury (TBI) affects millions of Americans each year and has been shown to disproportionately impact those subject to greater disparities in health. Female sex is one factor that has been associated with disparities in health outcomes, including in TBI, but sex differences in biomarker levels and behavioral outcomes after TBI are underexplored. This study included participants with both blunt and blast TBI with majority rating their TBI as mild. Time since injury was 5.4 (2.0, 15.5) years for females and 6.8 (2.4, 11.3) years for males. The aim of this cross sectional study is to investigate the relationship between postconcussive, depression, and post-traumatic stress disorder (PTSD) symptoms, as well as health related quality of life (HRQOL), and the levels of glial fibrillary acidic protein (GFAP), total tau (t-tau), neurofilament light chain (NfL), and ubiquitin C-terminal hydrolase-L1 (UCH-L1). Behavioral outcomes were evaluated with the Neurobehavioral Symptom Inventory (NSI), Patient Health Questionnaire-9 (PHQ-9), PTSD Checklist- Civilian Version (PCL-C), short form (SF)-36, and plasma levels of total tau, GFAP, NfL, and UCHL-1 measured with the Simoa-HDX. We observed that females had significantly higher levels of GFAP and tau (*ps* < 0.05), and higher PHQ-9 scores, NSI total scores, NSI- vestibular, NSI-somatosensory, NSI-affective sub-scale scores (*ps* < 0.05)), than males. In addition, females had lower scores in HRQOL outcomes of role limitations due to emotional problems, vitality, emotional well-being, social functioning, and pain compared to males (*ps* < 0.05). Correlation analysis showed positive associations between levels of tau and the NSI-total and NSI-cognitive sub-scale scores (*ps* < 0.05) in females. No significant associations were found for NfL or GFAP with NSI scores. For female participants, negative correlations were observed between tau and NfL concentrations and the SF-36 physical function subscale (*ps* < 0.05), as well as tau and the social function subscale (*p* < 0.001), while GFAP levels positively correlated with role limitations due to emotional problems (*p* = 0.004). No significant associations were observed in males. Our findings suggest that sex differences exist in TBI-related behavioral outcomes, as well as levels of biomarkers associated with brain injury, and that the relationship between biomarker levels and behavioral outcomes is more evident in females than males. Future studies are warranted to corroborate these results, and to determine the implications for prognosis and treatment. The identification of candidate TBI biomarkers may lead to development of individualized treatment guidelines.

## Introduction

Traumatic brain injury (TBI) affects over one million Americans each year and may lead to long-term debilitating behavioral symptoms particularly in groups with greater health disparities (Center for Disease Control and Prevention, National Center for Injury Prevention and Control CDC, 2021). Sex differences in TBI behavioral outcomes have been suggested, but possible molecular underpinnings are unclear. Current TBI treatment guidelines remain largely based on the studies with predominantly male participants (Biegon 2021). Exploring TBI outcomes and potential candidate TBI biomarkers based on sex differences might lead to development of precision medicine therapies.

In a recent review, studies with mild to moderate TBI were more likely to use social-behavioral measures and women reported worse behavioral outcomes than men; however, for severe TBI, studies were more likely to use survival and community integration as outcome measures and women fared better than men (Gupte et al., 2019). In a review of sex differences in mild TBI, researchers found that females have a proportionally higher incidence of concussions in sports; however, comparisons of symptom prevalence between the sexes have had mixed results (Merritt et al., 2019). Other reviews highlight the paucity of studies evaluating sex differences in blood-based biomarkers in TBI (Strathmann et al., 2014; Gupte et al., 2019).

Commonly measured biomarkers of TBI include total tau, neurofilament light chain (NfL), glial fibrillary acidic protein (GFAP), and ubiquitin C-terminal hydrolase-L1 (UCH-L1) (Kawata et al., 2016; Shahim et al., 2020a). In brain tissue of rats, researchers reported that both sexes had upregulation in gene expressions of GFAP, myelin basic protein, and tau following TBI, and while higher levels of GFAP was observed in female rats, in male rats it was significantly decreased (Wright et al., 2017). No sex differences in the biomarkers were noted in separate murine study post severe closed head injury (Rubenstein et al., 2017a). A recent study that examined 20 female and 20 male athletes found that plasma tau levels were significantly higher in females 6 h and 3 days after a concussive event than in males (Mondello et al., 2020). Accumulating evidence suggests that, not only may sex differences exist, but an exploration of outcomes and associations with biomarkers are paramount to furthering the current state of the science.

The aims of this project are to examine sex differences in postconcussive, depressive, and post-traumatic stress disorder (PTSD) symptoms, levels of circulating GFAP, tau, NfL, and the relationship between symptoms and biomarkers as well as health-related quality of life (HRQOL).

## Materials and Methods

### Study Design

The study used a cross-sectional design. Participants (*n* = 289) were enrolled in a larger ongoing study at two locations: Fort Belvoir Community Hospital (FBCH, Virginia) and Walter Reed National Military Medical Center (WRNMMC, Bethesda, Maryland). The participants include either active-duty or retired military service members as well as civilians without prior military experience. The participants underwent a medical history and physical exam and had blood samples collected. They also completed a series of behavioral symptom questionnaires. The exclusion criteria in this study included prior diagnosis of psychosis, schizophrenia, schizoaffective disorder, bipolar disorder, conversion disorder, or personality disorder. Institutional review board (IRB) approvals were obtained from the Uniformed Services University of the Health Sciences (USUHS), FBCH, and WRNMMC. Prior to data and sample collection, a written witnessed informed consent was collected from each research participant. Participants with complete blood, clinical, and behavioral outcomes were included in a convenience sample for this analysis.

To characterize the lifetime TBI history of each participant, we used the Ohio State University Traumatic Brain Injury Identification Method (OSU TBI-ID), which is a structured interview carried out by research staff. This interview assesses the number of TBIs that each participant sustained in their lifetime. For each TBI, the OSU TBI-ID reports on age at injury, cause of injury, incidence of loss of consciousness (LOC), and whether the participant was dazed and/or experienced a memory gap. A positive TBI was defined as an insult to the head that resulted in LOC and/or absence of concussion. Participants also reported length of the time they lost consciousness as part of OSU assessment. Because participants had multiple TBIs, the most recent TBI and most significant (higher severity on LOC) were used for the classification of self-reported severity. Self-reported LOC and AOC were used to classify the severity into mild (AOC and/or LOC of zero to 30 min), moderate (LOC greater than 30 min and less than 24 h), and severe (LOC greater than 24 h) (The Management of Concussion-Mild TBI Working Group, 2009). Additional neuroimaging or post-traumatic amnesia were not available for classification at the time of data collection. Time since last injury (TSI) was defined as the difference between the date of last known TBI and the date of blood collection.

### Blood-Based Biomarkers

Plasma were separated from peripheral blood collected from study participants following laboratory standard operating procedures and stored at −80°C until further analysis. Protein biomarker measurement was performed on the Simoa HD-x Analyzer (Quanterix Corperation, Lexington, MA), which allows for multiplex detection at single protein molecule level. Single protein molecules are captured and labeled on beads using standard ELISA reagents. After fluorescent labeling of the proteins, the Simoa HD-x Analyzer enables protein concentrations to be determined digitally. By isolating and detecting single immunocomplexes in arrays of femtoliter-volume wells, digital ELISA enables clinically important proteins in serum to be measured at sub-femtomolar concentrations that allows peripheral samples of blood to approximate central activity of neuropeptides. The Simoa Human Neurology 4-Plex Assay (N4PA, Cat# 102153, Quanterix Corperation, MA) measures four important neurology biomarkers detected in cerebrospinal fluid (CSF) and blood. The four targets are total tau, NfL, GFAP, and UCH-L1. The recommended low limit of quantifications (LLOQs) for tau, NfL, and GFAP in plasma are 0.212, 0.964, and 1.868 pg/ml, respectively. All measurements obtained in this study were within the assay quantification range. UCH-L1™ and Banyan GFAP™ are registered trademarks of Banyan Biomarkers. In total 29 samples (23 samples for tau, six samples for NfL) with intra-sample variability of more than 20.4% were excluded from the further analysis (Supplementary Table S1).

UCHL-1 results had CVs greater than 20% in about 59% of total samples and this biomarker was excluded from statistical analysis. The half-life of UCH-L1 is <12 h and it declines after a day to several days thereafter depending on the severity of the injury (Diaz-Arrastia et al., 2014). Because our samples are chronic, it is likely that high CVs in the UCH-L1 dataset were due to having many samples at the LLOQ of this assay. Research staff processing blood-based biomarkers were blind to the male and female groups.

### Behavioral Outcomes

Postconcussive symptoms were measured using the Neurobehavioral Symptom Inventory (NSI), a 22-item questionnaire that includes somatosensory, affective, cognitive, and vestibular sub-scales. The questionnaire has been validated in the TBI population with an acceptable test-retest indices (*r* ranging 0.78–0.94) and good internal consistency (*α* ≥ 0.80) for NSI total, and test-retest reliability with *r* ranging 0.52 to 0.91 and *α* ≥ 0.80 for its subscales (Silva 2020). The items on the questionnaire are rated from 0 (none) to 4 (severe) with higher scores indicating worse symptoms.

Depression symptom severity was measured using a 9-item Patient Health Questionnaire (PHQ-9) that has excellent sensitivity (0.93) and specificity (0.89) and test-retest reliability (*r* = 0.76, ĸ = 0.46) (Fann et al., 2005). It uses a Likert scale rated from “not at all” to “nearly every day” for each of the 9 items, so that the total score can range from 0 to 27, with higher scores constituting greater severity of depression (Kroenke et al., 2001).

Posttraumatic stress symptom severity was measured with the PTSD Checklist- Civilian Version (PCL-C), a 17-item self-reported questionnaire featuring a Likert scale rated from “not at all” to “extremely” (Ruggiero et al., 2003) on each item. The questionnaire has been previously validated and shown to have high internal consistency for PCL total (*α* = 0.94) as well as convergent validity (*r* > 0.75) (Ruggiero et al., 2003). The total score ranges from 17 to 80, where higher scores indicate greater symptom burden.

Health-related quality of life (HRQOL) was measured using the short form (SF)-36 with previously established excellent reliability (≥0.76) and acceptable validity (*r* ≥ 0.40) (Ware and John 2000). The questionnaire evaluates physical and mental HRQOL and is divided into eight subscales: physical functioning, role-physical, bodily pain, general health, vitality, social functioning, role-emotional, and mental health. The lower scores indicate lower HRQOL and higher scores indicate better HRQOL (Ware et al., 2001).

### Statistical Methods

All statistical analyses were performed using SPSS Grad Pack 24.0 and figures were created in GraphPad Prism (version 9.0.0). Demographic, clinical, and biomarker variables were non normally distributed, thus nonparametric test statistics of Mann Whitney *U* test for continuous variables and Pearson chi -square test or Fisher’s exact test for categorical variables were run to assess group differences between females and males. Binominal logistic regression models were run to control for significant demographic covariates. We performed log base two transformations to decrease skewness of the blood-based biomarkers to improve fit of the logistic regression models. We also ran spearman correlation analyses between biomarker levels and behavioral and HRQOL scores. A simple linear regression line was fit to the correlation data in GraphPad Prism.

## Results

### Demographic and Clinical Characteristics

Demographic, behavioral, and clinical characteristics of 289 TBI participants are presented in Table 1. The sample included 61 female and 228 male participants. The significantly different variables between the groups were age, BMI, race, military status, and highest education completed. Female participants were older (median = 42.0 years, IQR = 33.0–54.5) compared to male participants (median = 38.0, IQR = 32.0–47.0) (*p* = 0.030). Female participants had lower BMI (median = 27.0, IQR 24.0–29.5) compared to male participants (median = 28.0, IQR 26.0–31.0) (*p* = 0.008). Over half, 36 (59.0%) of female participants were White and 13 (21.3%) were Black or African American, while male participants were predominantly White 183 (80.3%) (*p* = 0.003). Twenty-three female participants (37.7%) were active duty service members compared to 169 (74.1%) male; 12 (19.7%) female participants reported no prior or current military service compared to 2 (0.9%) male (*p* < 0.001). Over a third, 24 (39.3%) female participants had graduate degree compared to 63 (27.6%) male participants, while 14 (22.9%) females obtained high school diploma compared to 81 (35.5%) male (*p* = 0.004). Majority of participants in both groups reported mild TBI (mTBI) using both most recent and most significant events. While most of the females (60.7%) had blunt force TBI, males had reported both blunt and blast TBI (68.4%) (Table 1).

**TABLE 1:** Demographic and clinical variables.

Characteristic	Overall (289^a^)	Female (61^a^)	Male (228^a^)	p^b^
Age	38.0 (32.0, 48.0)	42.0 (33.0, 54.5)	38.0 (32.0, 47.0)	0.030*
BMI	28.0 (26.0, 31.0)	27.0 (24.0, 29.5)	28.0 (26.0, 31.0)	0.008*
Race				0.003*
White	219 (75.8%)	36 (59.0%)	183 (80.3%)	
Black or African-American	44 (15.2%)	13 (21.3%)	31 (13.6%)	
Asian	16 (5.5%)	7 (11.5%)	9 (3.9%)	
American Indian or Alaska Native	4 (1.4%)	3 (4.9%)	1 (0.4%)	
Unknown, unable to provide	4 (1.4%)	1 (1.6%)	3 (1.3%)	
Pacific Islander	2 (0.7%)	1 (1.6%)	1 (0.4%)	
Ethnicity				0.545
Not Hispanic or Latino	236 (81.7%)	52 (85.2%)	184 (80.7%)	
Hispanic or Latino	50 (17.3%)	9 (14.8%)	41 (18.0%)	
Unknown, unable to provide	3 (1.0%)	0 (0.0%)	3 (1.3%)	
Highest education				0.004*
GED/High School Diploma	84 (29.1%)	8 (13.1%)	76 (33.3%)	
Vocational training	11 (3.8%)	6 (9.8%)	5 (2.2%)	
Associate Degree	35 (12.1%)	9 (14.8%)	26 (11.4%)	
Bachelor’s Degree	72 (24.9%)	14 (23.0%)	58 (25.4%)	
Master’s Degree	74 (25.6%)	21 (34.4%)	53 (23.2%)	
Doctoral Degree	13 (4.5%)	3 (4.9%)	10 (4.4%)	
Military Status				<0.001**
Active duty military	192 (66.4%)	23 (37.7%)	169 (74.1%)	
Retired from military	54 (18.7%)	13 (21.3%)	41 (18.0%)	
No military service	14 (4.8%)	12 (19.7%)	2 (0.9%)	
Veteran	13 (4.5%)	7 (11.5%)	6 (2.6%)	
National Guard	9 (3.1%)	2 (3.3%)	7 (3.1%)	
Reserve component	6 (2.1%)	4 (6.6%)	2 (0.9%)	
Inactive reserve	1 (0.3%)	0 (0.0%)	1 (0.4%)	
Number of TBIs	4.0 (2.0, 7.0)	4.0 (2.0, 6.0)	4.0 (2.0, 7.7)	0.178
TBI type				<0.001
Blunt force	94.0 (32.5%)	37.0 (60.7%)	57.0 (25.0%)	
Blast	17.0 (5.88%)	2.0 (3.3%)	15.0 (6.6%)	
Blunt and blast	178.0 (61.6%)	22.0 (36.1%)	156.0 (68.4%)	
Most recent TBI severity				0.615
mTBI	268 (92.7%)	55.0 (91.7%)	213.0 (93.4%)	
moTBI	12.0 (4.2%)	3.0 (5.0%)	9.0 (3.9%)	
sTBI	9.0 (3.1%)	3.0 (5.0%)	6.0 (2.6%)	
Highest TBI severity				0.345
mTBI	242.0 (83.7%)	51.0 (85.0%)	191.0 (83.8%)	
moTBI	33.0 (11.4%)	5.0 (8.2and)	28.0 (12.3%)	
sTBI	14.0 (4.8%)	5.0 (8.2%)	9.0 (3.9%)	
TSI (years)	6.4 (2.3, 11.7)	5.4 (2.0, 15.5)	6.8 (2.4, 11.3)	0.900
PHQ9	7.0 (3.0, 12.0)	10.0 (4.0, 15.8)	6.5 (2.3, 12.0)	0.010*
NSI-total	22.0 (11.5, 37.0)	34.0 (16.5, 50.5)	21.0 (10.0, 34.0)	<0.001**
NSI vestibular	2.0 (0.0, 4.0)	2.0 (1.0, 5.0)	1.0 (0.0, 3.0)	0.002*
NSI somatosensory	5.0 (2.0, 10.0)	9.0 (4.0, 15.0)	4.5 (2.0, 9.0)	<0.001**
NSI cognitive	6 (2.5, 10.0)	8.0 (3.0, 11.0)	6.0 (2.0, 9.0)	0.065
NSI affective	8.0 (4.0, 13.0)	10.0 (6.0, 17.0)	7.5 (3.0, 12.8)	0.006*
PCL-C	34.0 (24.0, 49.0)	38.0 (24.5, 56.0)	32.0 (24.0, 47.0)	0.092
SF-36 subscales				
Physical Functioning	90.0 (70.0, 100.0)	85.0 (67.5, 95.0)	90.0 (70.0, 100.0)	0.138
Role Limitations Due to Physical Problems	50.0 (0.0, 100.0)	50.0 (0.0, 75.0)	50.0 (0.0, 100.0)	0.170
Role Limitations Due to Emotional Problems	67.0 (33.0, 100.0)	33.0 (0.0, 100.0)	67.0 (33.0, 100.0)	0.025*
Vitality	45.0 (25.0, 62.5)	30.0 (15.0, 60.0)	45.0 (25.0, 65.0)	0.024*
Emotional Well-Being	72.0 (52.0, 88.0)	64.0 (44.0, 80.0)	76.0 (56.0, 88.0)	0.019*
Social Functioning	75.0 (37.5, 100.0)	50.0 (25.0, 87.5)	75.0 (37.5, 100.0)	0.002*
Pain	55.0 (32.5, 80.0)	45.0 (22.5, 67.5)	57.5 (33.1, 80.0)	0.004*
General Health	60.0 (45.0, 80.0)	55.0 (40.0, 77.5)	65.0 (45.0, 83.8)	0.175

a Median (IQR); n (%).

b Mann Whitney *U* test; Pearson Chi -square test, Fisher’s exact test, p* <0.05, p**<0.001. Data are represented as median (IQR), or in the case of categorical variables, n (%)^1^.

Abbreviations: BMI, body mass index; TSI, time since injury; PHQ9, Patient Health Questionnaire 9; NSI, Neurobehavioral Symptom Inventory; PCL-C, PTSD Checklist Civilian Version; SF-36, Short Form 36 Health Survey Questionnaire; IQR, interquartile range, mTBI, mild TBI; moTBI, moderate TBI; sTBI, severe TBI.

### Biomarker Analysis

**FIGURE 1:**
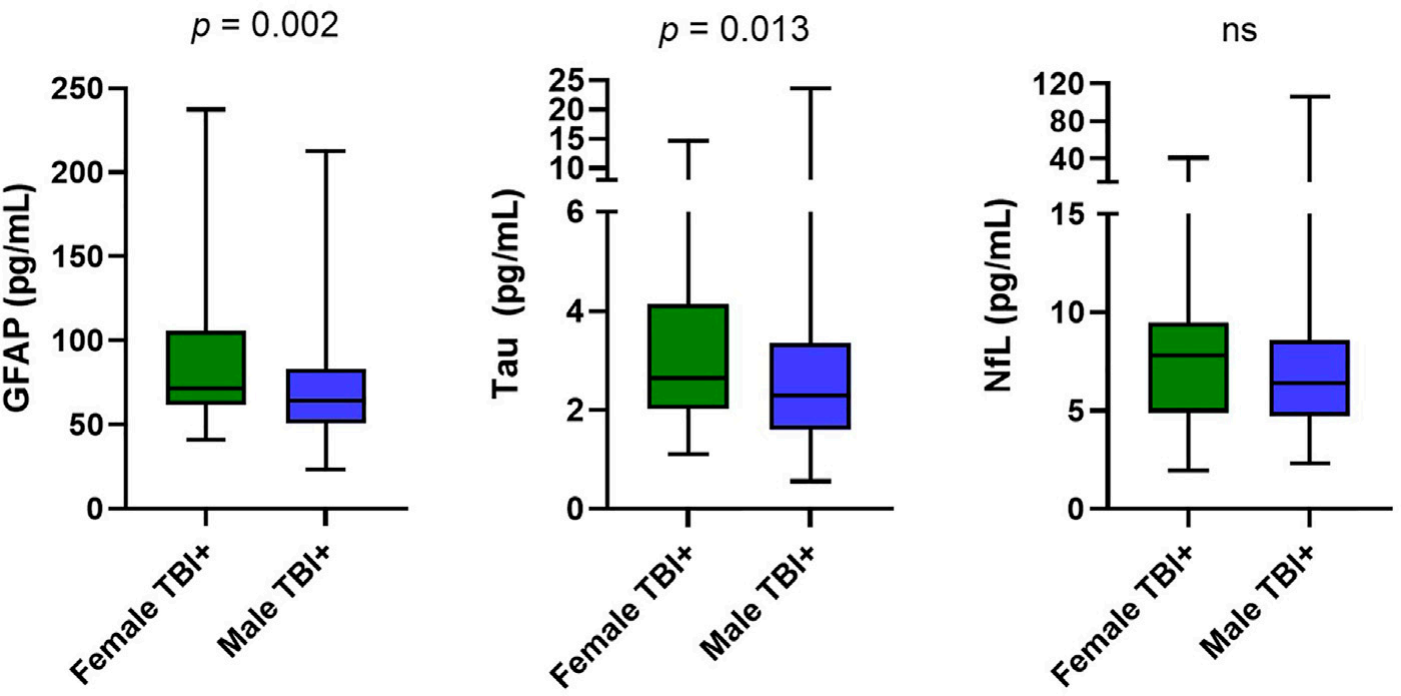
Group Differences in biomarkers. Distributions are represented via boxplots, showing median, IQR, and maximum and minimum. Mann-Whitney U results comparing two groups showed significantly higher GFAP (*p* = 0.002) levels in the female group (median = 71.7, IQR 62.0–106.0) compared to the male group (median = 64.2, IQR 50.7–83.0). Concentrations of t-tau (*p* = 0.013) were significantly elevated in the female group (median = 2.6, IQR 2.1–4.1) compared to the male group (median = 2.3, IQR 1.6–3.4). NfL in the female group (median = 7.8, IQR 4.9–9.4) did not differ significantly (*p* = 0.132) from the male group (median = 6.4, IQR 4.7–8.6). Abbreviations: GFAP, Glial fibrillary acidic protein; NfL, Neurofilament light chain; t-tau total tau.

Female participants had higher levels of GFAP (*p* = 0.002) and tau (*p* = 0.013) compared to male participants (Figure 1). After controlling for age, BMI, race, education, and military status in logistic regression models, t-tau (*p* = 0.018) and GFAP (*p* = 0.018) remained significantly different between groups (Table 2). NfL did not differ between female and male participants (*p* = 0.13). Hindered by the small sample size, biomarker data for controls is available in Supplementary Table S2.

**TABLE 2:** Logistic regression models controlling for significant covariates.

Female vs. Male participants			
	Predictors	Coefficient (B)	*p*
Plasma t-tau			
	Age	0.012	0.505
	BMI	0.144	0.001
	Race	−1.175	0.001
	Highest Education	0.578	0.164
	Military Status	−1.803	<0.001
	Plasma tau	−0.443	0.018
Plasma GFAP			
	Age	0.033	0.087
	BMI	0.130	0.003
	Race	−1.357	<0.001
	Highest Education	0.545	0.190
	Military Status	−1.790	<0.001
	Plasma GFAP	−0.759	0.018

Abbreviations: GFAP, Glial fibrillary acidic protein; t-tau, total tau

Logistic regression results controlling for covariates of age, BMI, race, education, and military status, show GFAP and t-tau remain significant.

### Behavioral Symptoms

We compared behavioral symptoms between female and male participants. PHQ-9 scores were significantly higher in female compared to male participants (*p* = 0.01). NSI total (*p* < 0.001), NSI- vestibular (*p* = 0.002), NSI-somatosensory (*p* < 0.001), and NSI-affective scores (*p* = 0.006) were also higher in females. No significant differences were found in NSI-cognitive subscale (Table 1). We also compared HRQOL between the female and male groups. Female participants had lower scores (lower HRQOL) in role limitations due to emotional problems (*p* = 0.025), vitality (*p* = 0.024), emotional well-being (*p* = 0.019), social functioning (*p* = 0.002), and pain (*p* = 0.004) compared to male participants (Table 1). There were no differences in SF-36 subscales of physical functioning, role limitations due to physical problems, and general health. We observed no group differences in PCL-C scores. Levels of biomarkers and behavioral symptoms remained significant after removing individuals without military status (Supplementary Table S3). The behavior data for control groups was limited by small sample size in females (n = 22) and is included in Supplementary Table S2.

### Correlations Between Biomarkers Levels and Behavioral Symptoms

**FIGURE 2:**
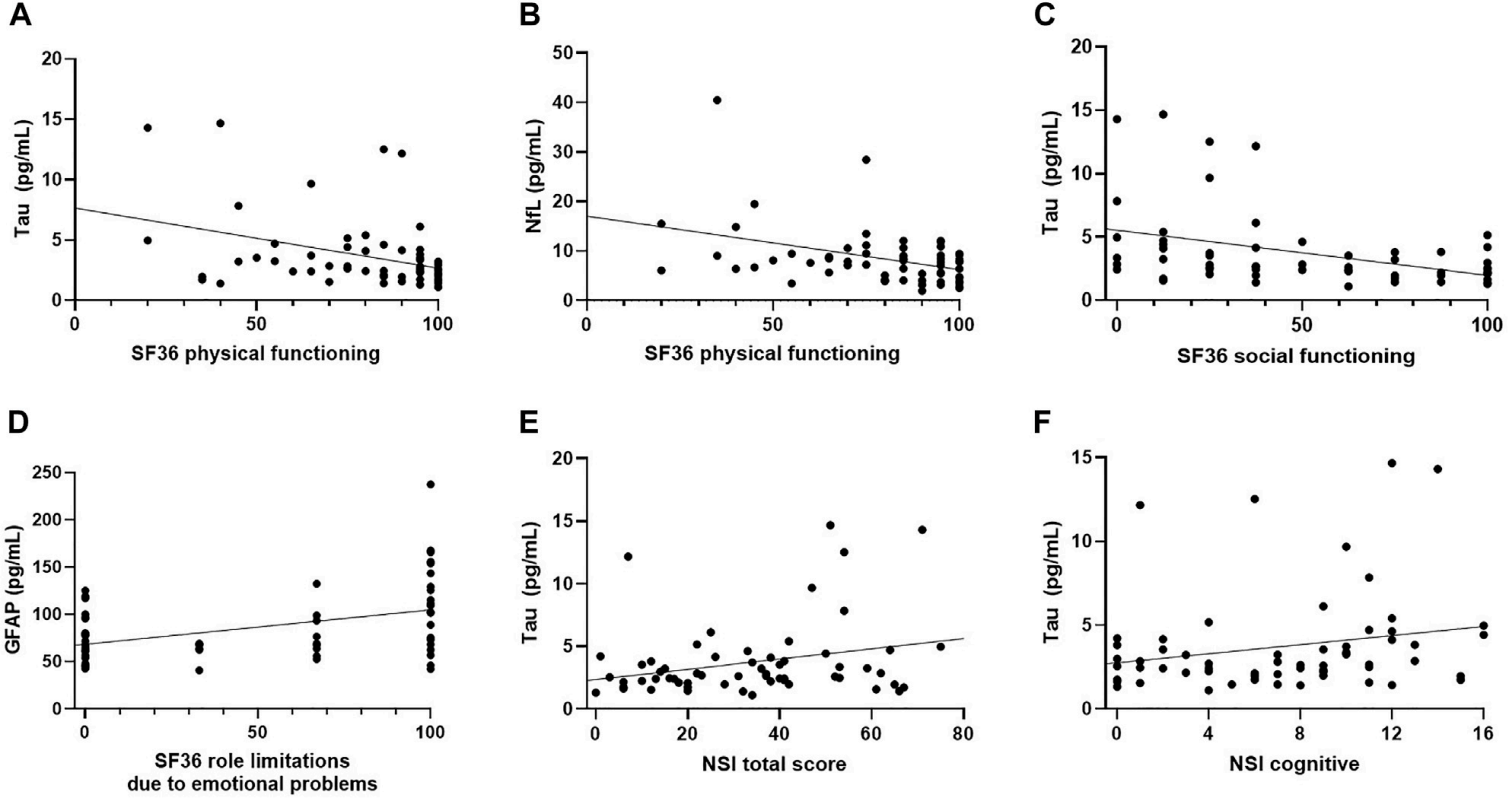
Scatter Plots of Significant Correlations in Female TBI Group. **(A)** T-tau negatively correlated with SF-36 physical functioning (*ρ* = −0.297, *p* = 0.020). **(B)** t-tau negatively correlated with SF-36 social functioning (*ρ* = −0.449, *p* < 0.001), **(C)** NfL negatively correlated with SF-36 physical functioning (*ρ* = −0.325, *p* = 0.011), **(D)** GFAP positively correlated with SF-36 subscale of role limitations due to emotional problems (*ρ* = 0.365, *p* = 0.004), **(E)** t-tau positively correlated with NSI-total (*ρ* = 0.255, *p* = 0.047), **(F)** t-tau had positive association with NSI-cognitive subscale (*ρ* = 0.263, *p* = 0.041).

In females, we observed positive correlations between levels of tau and NSI total (*ρ* = 0.255, *p* = 0.047) and NSI-cognitive subscale scores (*ρ* = 0.263, *p* = 0.041). Tau was also marginally significantly correlated with NSI somatosensory scores (*ρ* = 252, *p* = 0.050). No significant associations were found for NfL/GFAP and the NSI scores. For measures of HRQOL, in the female group, tau (*ρ* = −0.297, *p* = 0.020) and NfL (*ρ* = −0.325, *p* = 0.011) concentrations negatively correlated with SF-36 subscale of physical functioning. Tau was also inversely correlated with SF-36 subscale of social functioning (*ρ* = −0.449, *p* < 0.001) and GFAP had positive association with SF-36 subscale of role limitations due to emotional problems (*ρ* = 0.365, *p* = 0.004). No significant associations were observed for measures of behavioral symptoms and HRQOL in the male group (Figure 2, Supplementary Table S4).

## Discussion

To our knowledge, this study is the first to examine levels of circulating biomarkers and symptom scores in the context of sex differences in chronic TBI. In this cross-sectional convenience sample analysis, we found that levels of circulating plasma GFAP and tau were significantly higher in the female group compared to the male group. NfL levels did not differ significantly between groups. Behavioral symptoms of depression, NSI total, NSI-vestibular, NSI-somatosensory, and NSI- affective scores were higher in the female group as well. However, HRQOL of role limitations due to emotional problems, vitality, emotional well-being, social functioning, and pain were higher in the male group indicating better HRQOL. In addition, bivariate correlation results showed positive associations between symptoms, HRQOL scores and levels of GFAP, NfL, and tau.

We observed that levels of GFAP and tau were significantly higher in females. GFAP is an intermediate filament protein found in astrocytes of central nervous system and a marker of reactive astrocytes seen in neurodegenerative disease (Hol and Pekny 2015) and in TBI (Huang et al., 2015; McMahon et al., 2015). Reactive astrocytes can initiate pro-and anti-inflammatory immune mediators, communicate with innate and adaptive immune systems as well as neurons, glia, and vascular cells, and regulate blood-brain barrier in response to TBI (Burda et al., 2016). A recent study suggested that GFAP is a promising biomarker in distinguishing patients with acute mTBI from controls especially when combined with tau and NfL (Gill et al., 2018a). Another study reported that GFAP combined with UCHL-1 has excellent negative predictive value and sensitivity in predicting acute intracranial injuries detected by head computed tomography scans (Bazarian et al., 2018). In subacute and chronic TBI, serum GFAP levels have been shown to be elevated; however in contrast to acute mTBI, these levels were not sensitive in determining injury severity or outcomes (Shahim et al., 2020b). Few clinical studies have examined circulating biomarkers following TBI in relation to sex differences; however, several preclinical investigations have identified sexual dimorphic outcomes. Similar to our study, in mTBI, brain expression of GFAP was found to be elevated in female rats while being significantly lower in male rats (Wright et al., 2017). However, there were no sex differences in repetitive mTBI (Wright et al., 2017) or severe TBI in preclinical models (Rubenstein et al., 2017b).

In addition to GFAP, we also observed sex differences in tau following TBI. Tau is a microtubule-associated protein located on neuronal axons and its dysmetabolism plays a role in a number of neurodegenerative conditions such as Alzheimer’s disease (AD), corticobasal degeneration, as well as TBI (Weingarten et al., 1975; Olivera et al., 2015; Gill et al., 2018b; Didonna 2020). Plasma phosphorylated tau (p-tau) and p-tau-t-tau ratios have been demonstrated to distinguish patients with acute and chronic TBI from healthy controls (Rubenstein et al., 2017b). In chronic mTBI, exosomal tau and p-tau have been shown to be elevated in repetitive mTBI (Kenney et al., 2018). Our findings show that tau is significantly elevated in the female group compared to the male group after controlling for significant covariates. This is similar to findings in sports related concussions, where plasma tau was significantly higher in female athletes up to 3 days after initial injury (Mondello et al., 2020). Early tau depositions in the brain were higher in women measured by positron emission tomography in AD trajectory (Buckley et al., 2019). Higher levels of p-tau were also observed in women versus men among apolipoprotein E e4 (APOE4) carriers (Sundermann et al., 2020). Similarly, in a separate cohort, women had higher tau and p-tau concentrations in early stages of mild cognitive impairment (MCI) and subjective cognitive decline, while among non APOE e4 carriers, women had higher concentrations at the later stages of AD, dementia, and MCI (BabapourMofrad et al., 2020).

While we observed significant changes in GFAP and tau, NfL was not statistically significant between sexes in this report. NfL is 68 kDa subunit of the neurofilaments located on the neuronal cytoplasm which is released in response to CNS axonal damage due to neuroinflammation, neurodegeneration, and/or traumatic or vascular injury (Zetterberg 2016; Gaetani et al., 2019). Serum NfL has been shown to distinguish patients with mTBI, moderate or severe TBI for months and even years after injury (Shahim et al., 2020b). Cerebrospinal fluid NfL and CSF/serum albumin ratio were found to be significantly higher in male aging cohorts of AD (Skillbäck et al., 2021). These studies indicate there are sex differences in the levels of circulating biomarkers and additional studies are needed to corroborate these results.

Another interesting finding is that the female group had higher neurobehavioral and depressive symptom scores and significantly lower scores of HRQOL in role limitations due to emotional problems, vitality, emotional well-being, social functioning, and pain. We did not observe these trends in the male group. These results are consistent with previous reports of women having higher depressive symptoms and stress after mid-to moderate TBI (Bay et al., 2009) and higher likelihood of poor postconcussive symptom scores in mTBI (Bazarian et al., 2010). Our bivariate correlation analysis also showed that tau was positively associated with NSI total, NSI-cognitive, and marginally with NSI-somatosensory, while being negatively correlated with physical and social functioning of HRQOL. NfL also negatively correlated with physical functioning in the female group, indicating that as the concentrations of both tau and NfL increased the score for physical HRQOL functioning decreased. In our previous studies, we found that tau concentrations correlate significantly with postconcussive, post-traumatic, and depressive symptoms (Kenney et al., 2018; Pattinson et al., 2020) and exosomal NfL positively correlated with chronic postconcussive symptoms, PTSD, and depressive symptoms in predominantly male TBI groups (Guedes et al., 2020). In the current study, we also observed higher concentrations of GFAP to be associated with better emotional well-being HRQOL in the female group. While this result reached statistical significance, it is contradictory to the hypothesis that higher GFAP would be associated with lower HRQOL.

A possible mechanism underlying sex differences in TBI might include hormone levels. Several studies implicate the role of hormonal fluctuations during menstrual cycle in symptomatic presentations between sexes (Wunderle et al., 2014; Brown et al., 2015; Sundermann et al., 2020). For example, a study looking at the menstrual cycle phase and levels of progesterone concluded that women during the luteal phase of menstruation have significantly lower QOL metrics compared to women injured during the follicular phase (Wunderle et al., 2014). Lower testosterone levels resulted in higher p-tau concentrations in female APOE4 carriers, suggesting higher testosterone may be inversely related to p-tau levels in AD disease (Sundermann et al., 2020). Although we controlled for age (median age was 42 for women and 38 for men) in our analysis, hormonal fluctuations related to menopause have been documented to affect TBI outcomes. In large retrospective studies, peri- and post-menopausal women (age >50 years of age) had better outcomes and survival rate than men (Davis et al., 2006; Berry et al., 2009).

Another important factor to be considered is the role of gender as a fluid non-binary variable that may impact TBI experiences (Giordano et al., 2020). Societal gender expectations and individual vulnerabilities may affect symptoms and outcomes, coping skills, resources, and ultimately reintegration to society after TBI (Mollayeva et al., 2018). Currently, evidence is lacking on the effects of sex and gender in TBI research (Mollayeva et al., 2018) and often transgender groups are too small to be included in analysis (Giordano et al., 2020). Future clinical studies need to incorporate objective measures to further understand the impact of gender within sex on TBI outcomes.

This study has several limitations. First, our female participants in particular are of limited number. Future studies with larger sample sizes are warranted to corroborate our findings. Second, the injury mechanism of TBI (i.e., blast vs. non-blast TBI) may show a different effect on GFAP and total tau concentration. Future studies should investigate these biomarkers in relation to each injury type. Third, a cross-sectional design with self-reported TBI and symptoms makes it challenging to infer a causal relationship between the biomarkers and behavioral outcomes. Fourth, we did not measure hormonal differences or evaluated menstrual cycle phases. These may be important factors to include in future explorations on sex differences in TBI.

Lastly, the significance of the findings observed in GFAP and Tau between female and male participants in this study are affected by the lack of current clinical reference values for these biomarkers. Though GFAP and UCHL-1 have been shown to have high sensitivity for detection of acute TBI (Bazarian et al., 2018), the establishment of reference values in sex differences in TBI is still novel and clinical reference values are needed in the field. Reference ranges have been established for other biomarkers such as S100 calcium binding protein B (S100B) and neuron-specific enolase (NSE) and have been found to have sex-dependent differences. Specifically, higher s100B was observed in females and higher NSE in males (Hajduková et al., 2015). Several studies published blood-based reference ranges for GFAP and NfL; however, these ranges appear to differ between the studies (van Geel et al., 2002; Mayer et al., 2013). The utility of biomarkers, though promising in diagnostic and prognostic applications is limited by the lack of sex-based reference values. Additional studies are warranted to compare or refute clinical significance.

Despite these study limitations, this analysis shows robust sex differences in the levels of biomarkers and symptom burden in TBI after controlling for significant covariates (age, race, military status, BMI, and education). Our findings indicate that elevated biomarkers may be related to poor behavioral TBI outcomes and may impact overall HRQOL. Future explorations stratified by sex, gender, and age are paramount in delineating individual factors and identifying potential target biomarkers in treatment guidelines.

## Data Availability

The original contributions presented in the study are included in the article/Supplementary Files, further inquiries can be directed to the corresponding author.
