# Supplementary material for: Sex Differences in Behavioral Symptoms and the Levels of Circulating GFAP, Tau, and NfL in Patients With Traumatic Brain Injury
Source: Front Pharmacol. 2021 Nov 26;12:746491. doi: 10.3389/fphar.2021.746491 (PMC8662747; doi:10.3389/fphar.2021.746491)
Supplement: Supplementary file 1 [file DataSheet1.docx]

Supplementary Material

## Supplementary Table 1. List of excluded samples based on coefficient of variance (CV) of tau and NfL greater than 20.4%

| **Tau (pg/mL)** | **Tau CV** |
| --- | --- |
| 3.213 | 26.390 |
| 0.782 | 28.127 |
| 2.039 | 26.130 |
| 1.607 | 25.649 |
| 0.808 | 39.435 |
| 1.908 | 35.142 |
| 1.571 | 22.982 |
| 1.512 | 30.146 |
| 1.112 | 24.177 |
| 3.306 | 22.762 |
| 1.404 | 36.469 |
| 1.811 | 22.874 |
| 2.356 | 20.780 |
| 1.563 | 21.504 |
| 1.881 | 28.916 |
| 1.248 | 28.643 |
| 1.626 | 33.032 |
| 1.999 | 22.734 |
| 0.153 | 64.838 |
| 2.191 | 21.195 |
| 0.561 | 33.146 |
| 0.936 | 27.818 |
| 1.417 | 25.830 |
| **NfL (pg/mL)** | **NfL CV** |
| 9.274 | 21.745 |
| 5.105 | 21.771 |
| 5.847 | 21.806 |
| 3.378 | 25.346 |
| 2.205 | 43.107 |
| 3.095 | 23.650 |

No GFAP samples were excluded. Of the included samples with complete blood, clinical, and behavioral data, GFAP samples were within accepted range of intra sample variability of <20.4%

**Supplementary table 2**: Control group sex differences in members without TBI.

| **C**haracter**istic** | **Total N = 75** | **FEMALE**, N = 22*^1^* | **MALE**, N = 53*^1^* | ***p*-value***^2^* |
| --- | --- | --- | --- | --- |
| Age | 40.0 (29.0, 48.0) | 44.5 (34.0, 57.0) | 35.0 (29.0, 46.5) | 0.082 |
| BMI | 27.4 (25.1, 29.5) | 27.3 (25.0, 29.6) | 27.8 (25.4, 29.4) | 0.553 |
| Race |  |  |  | 0.849 |
| *White* | 53.0 (70.7) | 15.0 (68.2%) | 38.0 (71.7%) |  |
| *Black or African American* | 13.0 (17.3%) | 5.0 (22.7%) | 8.0 (15.1%) |  |
| *Asian* | 5.0 (6.7%) | 1.0 (4.5%) | 4.0 (7.5%) |  |
| *American Indian or Alaska Native/Inuit* | 2.0 (2.7%) | 1.0 (4.5%) | 1.0 (1.9%) |  |
| *Unknown, unable to provide* | 2.0 (2.6 %) | 0.0 | 2.0 (3.8%) |  |
| Ethnicity |  |  |  | 0.777 |
| *Not Hispanic or Latino* | 59.0 (78.7%) | 18.0 (81.8%) | 41.0 (77.4 %) |  |
| *Hispanic or Latino* | 15.0 (20%) | 4. (18.2%) | 11.0 (20.8%) |  |
| *Unknown, unable to provide* | 1.0 (1.3%) | 0.0 | 1.0 (1.9%) |  |
| Highest education |  |  |  | 0.011* |
| *GED or High School Diploma* | 18.0 (24.0%) | 1.0 (4.5%) | 17.0 (32.1%) |  |
| *Bachelor's Degree* | 15.0 (20.0%) | 9.0 (40.9%) | 6.0 (11.3%) |  |
| *Master's Degree* | 23.0 (30.7%) | 8.0 (36.4%) | 15.0 (28.3%) |  |
| *Associate's Degree* | 10.0 (13.3%) | 1.0 (4.5%) | 9.0 (17.0%) |  |
| *Doctoral Degree* | 5.0 (6.7%) | 1.0 (4.5%) | 4.0 (7.5%) |  |
| *Vocational Training, post High School* | 3.0 (4.0%) | 2.0 (9.1%) | 1.0 (1.9%) |  |
| *Vocational Training with no High School Diploma or GED* | 1.0 (1.3%) | 0.0 | 1.0 (1.9%) |  |
| Military status |  |  |  | <0.001** |
| *Active duty military* | 50.0 (66.7%) | 9.0 (40.9%) | 41.0 (77.4%) |  |
| *Retired from military* | 12.0 (16.0%) | 3.0 (13.6%) | 9.0 (17.0%) |  |
| *Veteran* | 4.0 (5.3%) | 4.0 (18.2%) | 0.0 |  |
| *National Guard* | 2.0 (2.7%) | 1.0 (4.5%) | 1.0 (1.9%) |  |
| *Reserve component* | 1.0 (1.3%) | None | 1.0 (1.9%) |  |
| *No military service* | 6.0 (8.0%) | 5.0 (22.7%) | 1.0 (1.9%) |  |
| PHQ9 | 2.0 (1.0, 4.0) | 2.0 (0.0, 7.5) | 2.0 (1.0, 4.0) | 0.497 |
| NSI-total | 7.0 (2.0, 13.0) | 9.5 (4.8, 16.5) | 6.0 (2.0, 13.0) | 0.214 |
| NSI vestibular | 0.0 (0.0, 1.0) | 0.0 (0.0, 2.0) | 0.0 (0.0, 1.0) | 0.161 |
| NSI somatosensory | 2.0 (0.0, 4.0) | 3.0 (2.0, 4.5) | 1.0 (0.0, 3.0) | 0.016* |
| NSI cognitive | 1.0 (0.0, 4.0) | 1.5 (0.0, 5.0) | 1.0 (0.0, 3.5) | 0.370 |
| NSI affective | 3.0 (1.0, 6.0) | 4.0 (1.8, 7.5) | 3.0 (1.0, 5.5) | 0.330 |
| PCL-C | 22.0 (19.0, 28.0) | 21.5 (19.0, 27.0) | 22.0 (18.0, 28.5) | 0.775 |
| **SF36 subscales** |  |  |  |  |
| Physical Functioning | 95.0 (90.0, 100.0) | 95.0 (83.8, 100.0) | 95.0 (90.0, 100.0) | 0.488 |
| Role Limitations Due to Physical Problems | 100.0 (75.0, 100.0) | 75.0 (18.8, 100.0) | 100.0 (75.0, 100.0) | 0.021* |
| Role Limitations Due to Emotional Problems | 100.0 (66.7, 100.0) | 100.0 (33.3, 100.0) | 100.0 (66.7, 100.0) | 0.164 |
| Vitality | 70.0 (55.0, 80.0) | 62.5 (33.8, 80.0) | 70.0 (57.5, 82.5) | 0.091 |
| Emotional Well-Being | 84.0 (76.0, 92.0) | 82.0 (72.0, 88.0) | 84.0 (80.0, 92.0) | 0.144 |
| Social Functioning | 100.0 (75.0, 100.0) | 100.0 (62.5, 100.0) | 100.0 (81.3, 100.0) | 0.286 |
| Pain | 80.0 (57.5, 100.0) | 72.5 (34.4, 90.0) | 80.0 (57.5, 100.0) | 0.119 |
| General Health | 80.0 (70.0, 90.0) | 80.0 (68.8, 85.0) | 85.0 (70.0, 90.0) | 0.329 |
| **Biomarkers** |  |  |  |  |
| GFAP | 67.1 (51.7, 96.8 | 84.9 (50.6, 128.7) | 63.7 (50.7, 84.4) | 0.094 |
| NfL | 6.6 (4.4, 9.2) | 8.1 (5.8, 10.9) | 5.6 (4.2, 7.7) | 0.025* |
| Tau | 2.2 (1.5, 3.2) | 2.0 (1.6, 2.9) | 2.3 (1.5, 3.6) | 0.376 |

*^1^*Median (IQR); n (%)

*^2^*Mann Whitney U test; Pearson's Chi-squared test

**Abbreviations**: BMI, body mass index; PHQ9, Patient Health Questionnaire 9; NSI, Neurobehavioral Symptom Inventory; PCL-C, PTSD Checklist Civilian Version; SF-36, Short Form 36 Health Survey Questionnaire; IQR, interquartile range.

Similar to TBI groups, females without TBI history had higher NSI-somatosensory compared to males without TBI. While most of the HRQOL scores did not differ, males had higher scores in role limitations due to physical problems control groups. GFAP and tau did not significantly differ between sexes in the control groups, however NfL was higher in females compared to males without TBI (*p*=0.16) (Supplementary Table 4). While these findings were interesting, the sex differences in TBI groups compared were much more pronounced. The small sample size in females (n=22) hindered our ability for robust statistical analysis.

**Supplementary table 3**: Sex differences in service members with TBI*

| **Characteristic** | **Overall**, N = 275*^1^* | **FEMALE**, N = 49*^1^* | **MALE**, N = 226*^1^* | **p-value***^2^* |
| --- | --- | --- | --- | --- |
| Age | 38.0 (32.0, 47.0) | 41.0 (32.5, 52.0) | 38.0 (32.0, 47.0) | 0.214 |
| BMI | 28.0 (26.0, 31.0) | 27.0 (24.5, 29.0) | 28.0 (26.0, 31.0) | 0.013* |
| Race |  |  |  | <0.001** |
| *White* | 210 (76.4%) | 28 (57.1%) | 182 (80.5%) |  |
| *Black or African-American* | 42 (15.3%) | 11 (22.4%) | 31 (13.7%) |  |
| *Asian* | 14 (5.1%) | 6 (12.2%) | 8 (3.5%) |  |
| *American Indian or Alaska Native/Inuit* | 4 (1.5%) | 3 (6.1%) | 1 (0.4%) |  |
| *Native Hawaiian or Other Pacific Islander* | 2 (0.7%) | 1 (2.0%) | 1 (0.4%) |  |
| *Unknown, unable to provide* | 3 (1.1%) | 0 (0.0%) | 3 (1.3%) |  |
| Ethnicity |  |  |  | 0.569 |
| *Not Hispanic or Latino* | 224 (81.5%) | 42 (85.7%) | 182 (80.5%) |  |
| *Hispanic or Latino* | 48 (17.5%) | 7 (14.3%) | 41 (18.1%) |  |
| *Unknown, unable to provide* | 3 (1.1%) | 0 (0.0%) | 3 (1.3%) |  |
| *Highest education* |  |  |  | 0.053 |
| GED or High School Diploma | 81 (29.5%) | 7 (14.3%) | 74 (32.7%) |  |
| *Bachelor's Degree* | 70 (25.5%) | 12 (24.5%) | 58 (25.7%) |  |
| *Master's Degree* | 69 (25.1%) | 16 (32.7%) | 53 (23.5%) |  |
| *Associate's Degree* | 33 (12.0%) | 7 (14.3%) | 26 (11.5%) |  |
| *Doctoral Degree* | 13 (4.7%) | 3 (6.1%) | 10 (4.4%) |  |
| *Vocational Training, post High School* | 9 (3.3%) | 4 (8.2%) | 5 (2.2%) |  |
| Military status |  |  |  | <0.001** |
| *Active duty military* | 192 (69.8%) | 23 (46.9%) | 169 (74.8%) |  |
| *Retired from military* | 54 (19.6%) | 13 (26.5%) | 41 (18.1%) |  |
| *Veteran* | 13 (4.7%) | 7 (14.3%) | 6 (2.7%) |  |
| *National Guard* | 9 (3.3%) | 2 (4.1%) | 7 (3.1%) |  |
| *Reserve component* | 6 (2.2%) | 4 (8.2%) | 2 (0.9%) |  |
| *Inactive reserve* | 1 (0.4%) | 0 (0.0%) | 1 (0.4%) |  |
| Number of TBIs | 4.0 (2.0-7.0) | 4.0 (2.0-6.0) | 4.0 (2.0-7.2) | 0.173 |
| TSI | 6.8 (2.3, 11.5) | 5.5 (2.0, 14.5) | 6.8 (2.5, 11.3) | 0.891 |
| PHQ9 | 7.0 (3.0, 12.0) | 10.0 (4.0, 16.5) | 6.5 (2.8, 12.0) | 0.009* |
| NSI-total | 22.0 (11.0, 37.0) | 34.0 (19.0, 52.5) | 21.0 (10.0, 34.0) | <0.001** |
| NSI vestibular | 2.0 (0.0, 4.0) | 3.0 (1.0, 5.0) | 1.0 (0.0, 3.0) | 0.001* |
| NSI somatosensory | 5.0 (2.0, 10.0) | 9.0 (5.0, 15.5) | 4.5 (2.0, 9.0) | <0.001** |
| NSI cognitive | 6.0 (3.0, 10.0) | 8.0 (3.5, 11.0) | 6.0 (2.0, 9.0) | 0.051 |
| NSI affective | 8.0 (4.0, 13.0) | 11.0 (6.0, 18.5) | 7.5 (3.0, 13.0) | 0.003* |
| PCL-C | 34.0 (24.0, 49.0) | 40.0 (26.0, 56.5) | 32.0 (24.0, 47.3) | 0.042* |
| **SF36 subscales** |  |  |  |  |
| Physical Functioning | 90.0 (70.0, 100.0) | 85.0 (65.0, 95.0) | 90.0 (70.0, 100.0) | 0.041* |
| Role Limitations Due to Physical Problems | 50.0 (0.0, 100.0) | 25.0 (0.0, 75.0) | 50.0 (0.0, 100.0) | 0.077 |
| Role Limitations Due to Emotional Problems | 67.0 (33.0, 100.0) | 33.0 (0.0, 100.0) | 67.0 (33.0, 100.0) | 0.007* |
| Vitality | 45.0 (25.0, 60.0) | 30.0 (15.0, 60.0) | 45.0 (25.0, 65.0) | 0.006* |
| Emotional Well-Being | 68.0 (52.0, 88.0) | 60.0 (42.0, 76.0) | 76.0 (56,0, 88.0) | 0.004* |
| Social Functioning | 75.0 (37.5, 100.0) | 37.5 (12.5, 81.3) | 75.0 (37.5, 100.0) | <0.001** |
| Pain | 55.0 (32.5, 80.0) | 42.5 (22.5, 58.8) | 57.5 (34.4, 80.0) | <0.001** |
| General Health | 60.0 (45.0, 80.0) | 55.0 (40.0, 75.0) | 65.0 (45.0, 81.3) | 0.076 |
| **Biomarkers** |  |  |  |  |
| GFAP | 65.1 (52.4, 84.3) | 68.0 (60.7, 97.0) | 64.2 (50.8, 83.2) | 0.036* |
| NfL | 6.5 (4.8, 8.7) | 7.6 (5.1, 9.5) | 6.4 (4.8, 8.6) | 0.275 |
| Tau | 2.4 (1.7, 3.5) | 2.7 (2.1, 4.2) | 2.3 (1.6, 3.4) | 0.012* |

*For the analysis, all participants with no prior military experience were removed (n = 275).

*^1^*Median (IQR); n (%)

*^2^*Wilcoxon rank sum test; Fisher's exact test; Pearson's Chi-squared test

**Supplementary table 4:** Spearmen correlation values of circulating biomarkers and behavioral outcomes.

|  | TBI+ Female | | | | | | TBI+ Male | | | | | |
| --- | --- | --- | --- | --- | --- | --- | --- | --- | --- | --- | --- | --- |
|  | Tau | | GFAP | | NfL | | Tau | | GFAP | | NfL | |
|  | Corr. | P | Corr. | P | Corr. | P | Corr. | P | Corr. | P | Corr. | P |
| NSI total | **0.255*** | 0.047 | -0.145 | 0.265 | 0.008 | 0.951 | -0.028 | 0.670 | 0.067 | 0.314 | 0.051 | 0.447 |
| NSI vestibular | 0.174 | 0.181 | -0.001 | 0.992 | 0.022 | 0.864 | -0.112 | 0.093 | 0.040 | 0.546 | 0.075 | 0.258 |
| NSI somatosensory | 0.252 | 0.050 | -0.185 | 0.154 | -0.094 | 0.473 | -0.028 | 0.673 | 0.068 | 0.308 | 0.045 | 0.503 |
| NSI cognitive | **0.263*** | 0.041 | -0.131 | 0.314 | 0.006 | 0.966 | -0.026 | 0.700 | 0.050 | 0.448 | 0.031 | 0.646 |
| NSI affective | 0.163 | 0.209 | -0.077 | 0.555 | 0.080 | 0.538 | 0.026 | 0.694 | 0.068 | 0.305 | 0.031 | 0.646 |
| PCL-C | 0.175 | 0.177 | -0.073 | 0.575 | 0.020 | 0.876 | 0.001 | 0.994 | 0.030 | 0.650 | 0.019 | 0.779 |
| PHQ9 | 0.173 | 0.185 | -0.111 | 0.397 | 0.090 | 0.496 | -0.019 | 0.776 | 0.004 | 0.948 | -0.004 | 0.957 |
| **SF36 subscales** |  |  |  |  |  |  |  |  |  |  |  |  |
| Physical Functioning | **-0.297*** | 0.020 | -0.216 | 0.095 | **-0.325*** | 0.011 | -0.011 | 0.871 | 0.057 | 0.395 | -0.043 | 0.521 |
| Role Limitations Due to Physical Problems | -0.207 | 0.109 | 0.153 | 0.240 | -0.079 | 0.543 | -0.016 | 0.814 | -0.007 | 0.920 | -0.059 | 0.374 |
| Role Limitations Due to Emotional Problems | -0.227 | 0.078 | **0.365**** | 0.004 | 0.098 | 0.453 | 0.007 | 0.913 | -0.010 | 0.883 | -0.104 | 0.117 |
| Vitality | -0.146 | 0.260 | 0.225 | 0.082 | -0.004 | 0.976 | -0.027 | 0.683 | -0.096 | 0.149 | 0.015 | 0.826 |
| Emotional Well-Being | -0.146 | 0.263 | 0.101 | 0.440 | -0.094 | 0.473 | -0.016 | 0.807 | -0.001 | 0.990 | 0.024 | 0.714 |
| Social Functioning | **-0.449**** | < 0.001 | 0.164 | 0.206 | -0.023 | 0.863 | 0.078 | 0.241 | -0.073 | 0.270 | -0.069 | 0.302 |
| Pain | -0.135 | 0.298 | 0.136 | 0.295 | -0.057 | 0.663 | 0.004 | 0.958 | -0.015 | 0.827 | -0.094 | 0.158 |
| General Health | -0.205 | 0.113 | 0.025 | 0.850 | -0.039 | 0.763 | -0.012 | 0.860 | -0.087 | 0.190 | -0.104 | 0.118 |

Supplementary table 2: rho value and p value for each correlation are represented in this table (n = 289)

*correlation is significant at the 0.05 level (2-tailed)

**correlation is significant at the 0.01 level (2-tailed)
